# Association of Non-Pharmaceutical Interventions to Reduce the Spread of SARS-CoV-2 With Anxiety and Depressive Symptoms: A Multi-National Study of 43 Countries

**DOI:** 10.3389/ijph.2022.1604430

**Published:** 2022-03-03

**Authors:** Kira E. Riehm, Elena Badillo Goicoechea, Frances M. Wang, Esther Kim, Luke R. Aldridge, Carly P. Lupton-Smith, Rachel Presskreischer, Ting-Hsuan Chang, Sarah LaRocca, Frauke Kreuter, Elizabeth A. Stuart

**Affiliations:** ^1^ Department of Mental Health, Johns Hopkins University, Baltimore, MD, United States; ^2^ Facebook, Menlo Park, CA, United States; ^3^ Joint Program in Survey Methodology, University of Maryland, College Park, MD, United States; ^4^ School of Social Sciences, University of Mannheim, Mannheim, Germany; ^5^ Statistical Methods Group, Institute for Employment Research, Nuremberg, Germany

**Keywords:** anxiety, COVID-19, depression, gender, non-pharmaceutical interventions, age

## Abstract

**Objectives:** To examine the association of non-pharmaceutical interventions (NPIs) with anxiety and depressive symptoms among adults and determine if these associations varied by gender and age.

**Methods:** We combined survey data from 16,177,184 adults from 43 countries who participated in the daily COVID-19 Trends and Impact Survey *via* Facebook with time-varying NPI data from the Oxford COVID-19 Government Response Tracker between 24 April 2020 and 20 December 2020. Using logistic regression models, we examined the association of [1] overall NPI stringency and [2] seven individual NPIs (school closures, workplace closures, cancellation of public events, restrictions on the size of gatherings, stay-at-home requirements, restrictions on internal movement, and international travel controls) with anxiety and depressive symptoms.

**Results:** More stringent implementation of NPIs was associated with a higher odds of anxiety and depressive symptoms, albeit with very small effect sizes. Individual NPIs had heterogeneous associations with anxiety and depressive symptoms by gender and age.

**Conclusion:** Governments worldwide should be prepared to address the possible mental health consequences of stringent NPI implementation with both universal and targeted interventions for vulnerable groups.

## Introduction

The Coronavirus Disease 2019 (COVID-19) pandemic has had a devastating impact on health and well-being around the world. To limit the spread of disease and mitigate the burden on health systems, non-pharmaceutical interventions (NPIs) have been rapidly adopted worldwide and include a range of restrictions, such as stay-at home orders, workplace closures, and social venue closures [1, 2]. Accumulating evidence has found that more stringent implementation of NPIs is highly effective at preventing COVID-19 infections, hospitalizations, and deaths [3–6]. However, some researchers have called for explicit consideration of the potential negative consequences of NPIs, including anxiety and depressive symptoms, high unemployment, deaths due to causes other than COVID-19 infection, and widening health inequities [7, 8]. With regards to mental health, some NPIs, most prominently stay-at-home orders, have been widely speculated to contribute to anxiety and depressive symptoms by restricting access to social support networks and inducing isolation [9, 10]. Females have been particularly vulnerable to adverse changes in mental health during the COVID-19 pandemic [11], which may be due to factors such as increased childcare responsibilities and disproportionate job loss as a result of school and workplace closures [12, 13]. Similarly, NPIs may also have more severe implications for mental health among young adults, who are overrepresented in the service industry and therefore more susceptible to job loss or wage cuts in response to restrictions [12].

Empirical studies of NPIs and mental health during the COVID-19 pandemic are scarce. At present, most studies are cross-sectional, collect data from single countries, and focus almost exclusively on stay-at-home orders instead of other NPIs [14–20]. In addition, to our knowledge, the extent to which gender and age moderate associations between NPIs and mental health has not been evaluated.

A clearer understanding of the association of NPIs with mental health is essential for informing ongoing surveillance efforts, designing preventive interventions, and preparing for potential outbreaks in the future. In this study, we combine data from a large, multi-national survey of adults from 43 countries with time-varying, national-level NPI data from April to December 2020 during the COVID-19 pandemic. Our objectives were to [1] examine the association of NPIs (i.e., stay-at-home orders, workplace closures, school closures, etc.) with anxiety and depressive symptoms among adults and [2] determine if these associations varied by gender and age. We hypothesized that different NPIs would display heterogeneous associations with anxiety and depressive symptoms, and that the magnitude of these associations would vary by gender and age.

## Methods

### Data Source

The COVID-19 Trends and Impact Surveys (CTIS) is a daily cross-sectional survey conducted by the Social Data Science Center at University of Maryland and the Delphi Group at Carnegie Mellon University in partnership with Facebook, Inc. [21]. On 23 April 2020, the international version of the survey was launched in over 200 countries and territories (Supplementary Text). The CTIS instrument was developed by experts in public health and survey methodology and includes the following sections: COVID-19 related symptoms, testing, contact history, preventive behavior, mental health, economic security, and basic demographics [21]. The questionnaire is publicly available and has been translated into 56 languages (listed in Supplementary Table S1) [22, 23].

The sampling methodology for CTIS is described elsewhere [24]. In short, the sampling frame is composed of daily active Facebook users who are at least 18 years old, residing in one of 200+ countries or territories, and using a supported language. With this coverage, greater than 95% of Facebook users are eligible. Each calendar day, the Facebook app invites a random sample, stratified using the administrative boundaries within countries or territories, to take the survey with an invitation posted on the News Feed [25]. After viewing the survey invitation, those interested in completing the survey are redirected to a survey administered by the partnering academic institutions on a website separate from Facebook. Facebook does not share or receive data from the academic partners other than a list of random identification numbers of those who completed the survey to calculate and share survey weights.

With regards to the weighting procedure, Facebook employs a two-stage weighting process with the goal of minimizing biases resulting from non-response and representativeness of the general population. First, the study sample is weighted to be more representative of the Facebook sampling frame with inverse propensity score weighting to adjust for non-response. Because Facebook receives a list of identification numbers that indicates who completed the survey with no additional details, the covariates used for the weighting procedure are gathered from internal Facebook data (age, gender, geographical variables, and other characteristics that have been found internally to predict survey response). Second, post-stratification equates the distribution of age and gender in the Facebook population to benchmarks from the United Nations Population Division 2019 World Population Projections. Additional details pertaining to the weighting methodology are available elsewhere [24].

### Participants

Our sample included participants ages 18 and over who responded to the CTIS from 24 April 2020 through 20 December 2020. Responses from the US version of the survey were not included because the items assessing mental health differed from those in the international survey. Because some of the 200+ countries and territories surveyed had comparatively small sample sizes and large variability in response rates, we limited our sample to countries that met at least one of the following criteria [1]: considered a member, candidate, or key partner of the Organisation for Economic Co-operation and Development (OECD) convention, or [2] had a sample size >600,000 during our study period. There were 43 countries that met at least one of these criteria and were included in analyses (Supplementary Text). Over the course of the study period within these 43 countries, 1,154,490,869 Facebook users saw the survey invitation and 20,033,237 (1.7%) responded to the survey, which is comparable to response rates from other social media-based surveys [26]. Missingness on the variables of interest ranged from 2 to 15% per variable, which resulted in 19.2% of the survey respondents missing data for at least one variable being excluded. The final analytic sample included 16,177,184 adults (16,083,027 for anxiety symptoms and 16,163,821 for depressive symptoms). The number of respondents per week across the study period is displayed in Supplementary Figure S1.

### Measures

#### COVID-19 Non-Pharmaceutical Interventions

**FIGURE 1:**
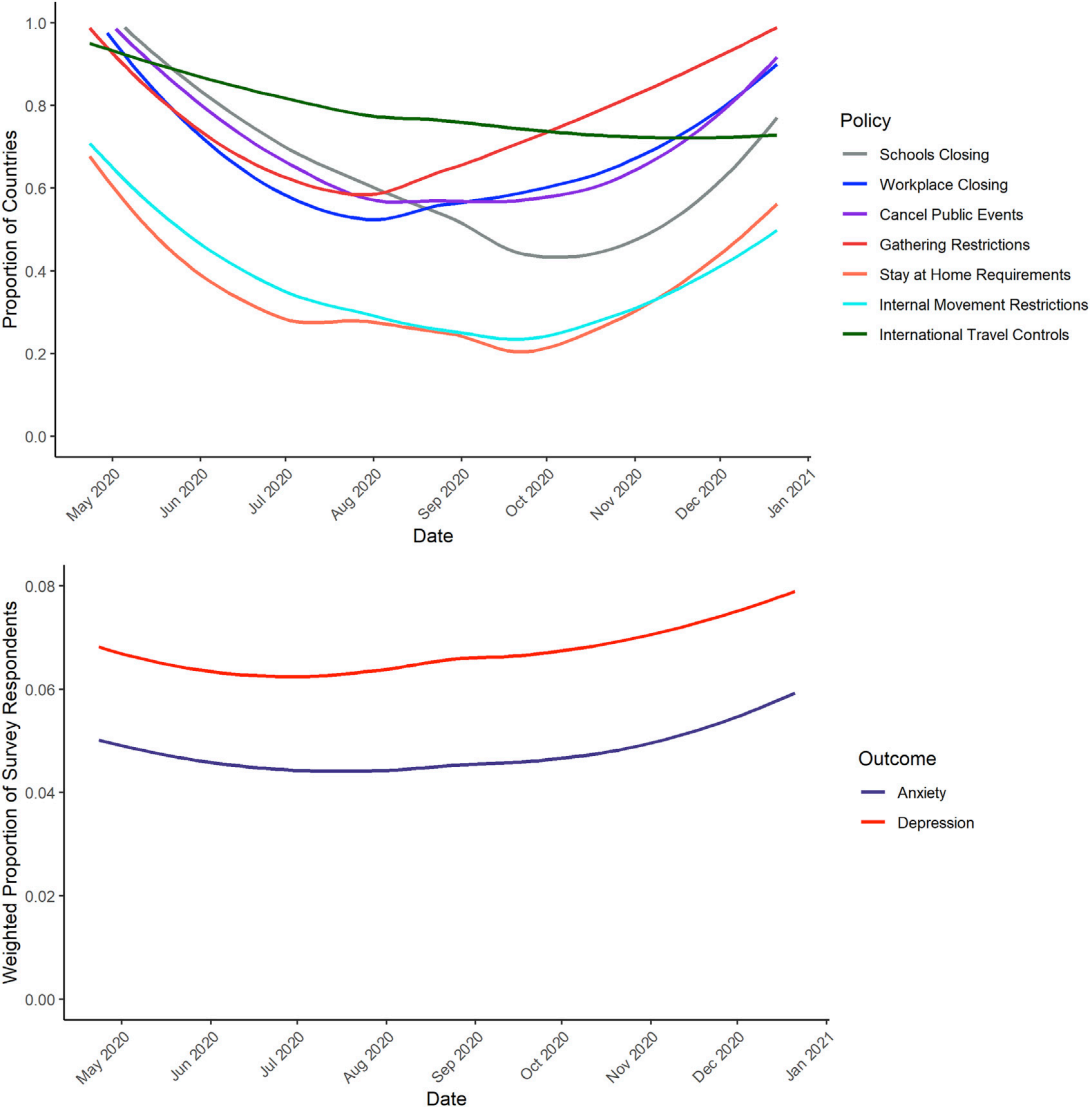
Temporal Trends in Policies [Panel **(A)**] and Prevalence of Anxiety and Depressive Symptoms [Panel **(B)**] (43 countries, 2020).

Data for containment policies were obtained from the Oxford COVID-19 Government Response Tracker (OxCGRT) [27]. Developed in response to the pandemic, the OxCGRT is an ongoing effort to compile data for a series of indicators of nationwide government responses. Data are collected by a team of over 100 students and staff from publicly available sources, including news articles and government briefings; the full methodology is described in depth elsewhere [28]. The database is updated on a day-to-day basis and the codebook is publicly available [27]. We examined the overall stringency index, which captures the strictness of government responses to the COVID-19 pandemic on a scale from 0 to 100, with higher scores indicating greater stringency/strictness. We also examined the following seven “containment and closure” NPIs for which there was sufficient temporal variation (Figure 1): school closures, workplace closures, cancellation of public events, restrictions on the size of gatherings, stay-at-home requirements, restrictions on internal movement, and international travel controls. We did not examine public transportation closures due to insufficient variation in implementation over time across countries.

We examined NPI implementation data from each country for each day of the study period (24 April 2020 to 20 December 2020). All policies, except for restrictions on gatherings and international travel controls, are measured on an ordinal scale with categories reflecting “no restriction,” “recommended restriction,” and “required restriction.” Because there was likely to be significant variation at sub-national levels within the “recommended restriction” category, we opted to dichotomize these indicators into “no restrictions/recommended restrictions” versus “required restrictions” to capture the “maximum effort” association of these NPIs, similar to other studies [6]. Restrictions on the size of gatherings were measured in terms of the number of people permitted to gather; we dichotomized this indicator into restrictions on gatherings of 101 or greater people versus restrictions on gatherings of 100 or fewer people. International travel controls were measured in terms of whether screenings or quarantines for arrival were required, or whether a full ban from some or all regions, or a full border closure, was in place. We dichotomized this variable into minimal travel restrictions (no restrictions, or only screening or quarantine required) versus stringent travel restrictions (travel bans present for some or all regions, or a full border closure).

#### Anxiety and Depressive Symptoms

Anxiety and depressive symptoms were measured with two items from the 10-item Kessler Psychological Distress Scale (K10) [29] that were included as part of the CTIS Survey. The K10 demonstrates adequate validity as a screening tool for serious psychological distress when compared to diagnostic interviews in the general population [30]. The K10 has also been translated into numerous languages and has been used cross-nationally as part of the World Health Organization (WHO) World Mental Health Surveys [31]. Participants were asked how often in the last 7 days they felt “so nervous that nothing could calm you down” (anxiety symptoms) and “so depressed that nothing could cheer you up” (depressive symptoms). Response options were “all of the time,” “most of the time,” “some of the time,” “a little of the time,” and “none of the time.” To capture a relatively homogenous group of participants with more severe symptoms, we dichotomized both items by collapsing “all of the time” and “most of the time” into one category and comparing to one category containing the other response options.

#### Country- and Individual-Level Covariates

We included covariates at both the country and individual levels that may be confounders of associations between the implementation of NPIs and anxiety and depressive symptoms. At the country level, we included the number of new COVID-19 cases and the number of new COVID-19 deaths per 1,000,000 people per day; these were treated as time-varying in analyses. We also included the economic support index from the OxCGRT as a time-varying covariate, which reflects the level of economic support provided to individuals within a country (i.e., income support and debt relief). At the individual level, we included age groups (18–24, 25–34, 35–44, 45–54, or 65+), gender (female or male), whether an individual was working outside the home (yes or no), and urbanicity (city or town/village/rural area).

### Statistical Analysis

#### Descriptive Analyses

We examined the distribution of individual-level variables among survey respondents. We also examined variation in NPI implementation, and the probability of reporting anxiety or depressive symptoms, across the survey period. We used locally smoothed regressions (span=0.75) to display trends in the proportions of countries with policy requirements and respondents demonstrating anxiety/depression symptoms over time.

#### Association of Overall Stringency Index and NPIs With Anxiety and Depressive Symptoms

We estimated associations between NPIs and the odds of reporting anxiety and depressive symptoms using logistic regression models. Statistical analyses were conducted in two stages and were adjusted for all country- and individual-level covariates listed above. First, we examined the association of the overall stringency index with anxiety and depressive symptoms. In these models, the overall stringency index was rescaled from 0–100 to 0–10 to improve interpretability. Second, we estimated a single model that included each of the seven NPIs of interest, as well as interaction terms between each NPI and gender, to examine moderation by gender. We followed the same procedure to examine moderation by age (dichotomized, 18–24 versus 25+). To account for temporal changes in anxiety and depressive symptoms across the pandemic, all models included fixed effects for survey month (April to December). To account for time-invariant characteristics of countries, all models included fixed effects for country (each of the 43 countries represented in the sample). All analyses included weights to account for survey non-response and increase representativeness of the general population. We used the results of the regression models to calculate the predicted probabilities of reporting anxiety and depressive symptoms, both in the presence and absence of a given NPI, with all other covariates set to their mean.

#### Sensitivity Analyses

We conducted additional analyses to test the sensitivity of our results to modelling assumptions. Across the study period, the NPIs of interest were moderately correlated (Supplementary Figure S2); to test sensitivity to multicollinearity, we compared the results of univariable models with each individual NPI to the results of our main multivariable model with all NPIs included simultaneously. We also tested whether an alternative dichotomization of the items measuring anxiety and depressive symptoms (“all of the time,” “most of the time,” and “some of the time” collapsed into a single category compared to another category containing the other response options) affected the pattern of results.

Statistical significance was assessed at *p* < 0.05. All analyses were conducted using R (R studio version 1.2.5042; R version 4.0.0). The COVID-19 Symptom Survey was reviewed and approved by the Institutional Review Board of the University of Maryland.

## Results

### Descriptive Analyses

Our sample included 16,083,027 survey respondents for anxiety symptoms and 16,163,821 respondents for depressive symptoms (Table 1). After weighting, females comprised 44% of the sample. Males and females were similar in their age distribution (males: 18–24=17.8%, 25–34=26.9%, 35–44=19.8%, 45–54=16.0%, 55–64=9.8%, 65+=9.6%; females: 18–24=16.3%, 25–34=26.7%, 35–44=18.5%, 45–54=16.4%, 55–64=9.7%, 65+=12.4%). Figure 1 depicts the variations in policies and anxiety/depression symptoms over time across all 43 countries. In general, NPI implementation tended to relax in the June–September 2020 months followed by reimplementation thereafter.

**TABLE 1:** Demographic Characteristics of COVID-19 Trends and Impact Survey Respondents (*n*=16,177,184, 43 countries, 2020).

Variable	Weighted Percentage (%)
Female	44.3
Age	
18–24	16.9
25–34	26.8
35–44	19.3
45–54	16.4
55–64	9.8
65+	10.8
Urban	53.1
Works Outside Home	35.2
Anxiety^a^	4.8
Depression^b^	6.8

Notes: ^a^N for anxiety symptoms is 16,083,027.

b N for depressive symptoms is 16,163,821.

### Association of Overall Stringency Index and NPIs With Anxiety and Depressive Symptoms

Results of the models with the overall stringency index are displayed in Table 2. For each ten-point increase in government response stringency, the odds of reporting anxiety symptoms increased by 1.4% (OR=1.014, 95% CI=1.008–1.019) and the odds of reporting depressive symptoms increased by 2.7% (OR=1.027, 95% CI=1.022–1.032). With regards to individual-level covariates, anxiety and depressive symptoms were more commonly reported by women compared to men, those in cities compared to town/village/rural areas, and those working outside the home compared to those working at home. The predicted probabilities of reporting anxiety and depressive symptoms across the range of the government response stringency index are plotted in Supplementary Figures S3, S4.

**TABLE 2:** Association of the Stringency Index with Anxiety (*n* = 16,083,027) and Depressive Symptoms (*n* = 16,163,821) Among Adults (43 countries, 2020).

Variable	Anxiety symptoms		Depressive symptoms	
	OR	95% CI	OR	95% CI
Stringency Index^a^	1.014	1.008, 1.019	1.027	1.022, 1.032
Economic Support Index^a^	0.987	0.984, 0.990	0.991	0.998, 0.994
Weekly COVID-19 cases^a^	1.001	0.990, 1.011	1.017	1.008, 1.027
Weekly COVID-19 deaths^a^	1.040	1.030, 1.051	1.019	1.009, 1.028
Female (ref = male)	1.489	1.475, 1.504	1.418	1.406, 1.431
Age in years (ref = 65+)				
18–24	4.428	4.318, 4.541	6.348	6.209, 6.490
25–34	3.147	3.070, 3.226	3.862	3.778, 3.948
35–44	2.411	2.352, 2.473	2.564	2.507, 2.623
45–54	1.919	1.870, 1.969	1.975	1.930, 2.021
55–64	1.444	1.403, 1.485	1.495	1.458, 1.534
Urban (ref = town/village/rural)	1.050	1.039, 1.062	1.071	1.061, 1.081
Works Outside Home (ref = no)	1.069	1.059, 1.080	0.977	0.968, 0.986

Note: Models include fixed effects for country and calendar time (month).

a Variables lagged to the week prior to survey date.

**FIGURE 2:**
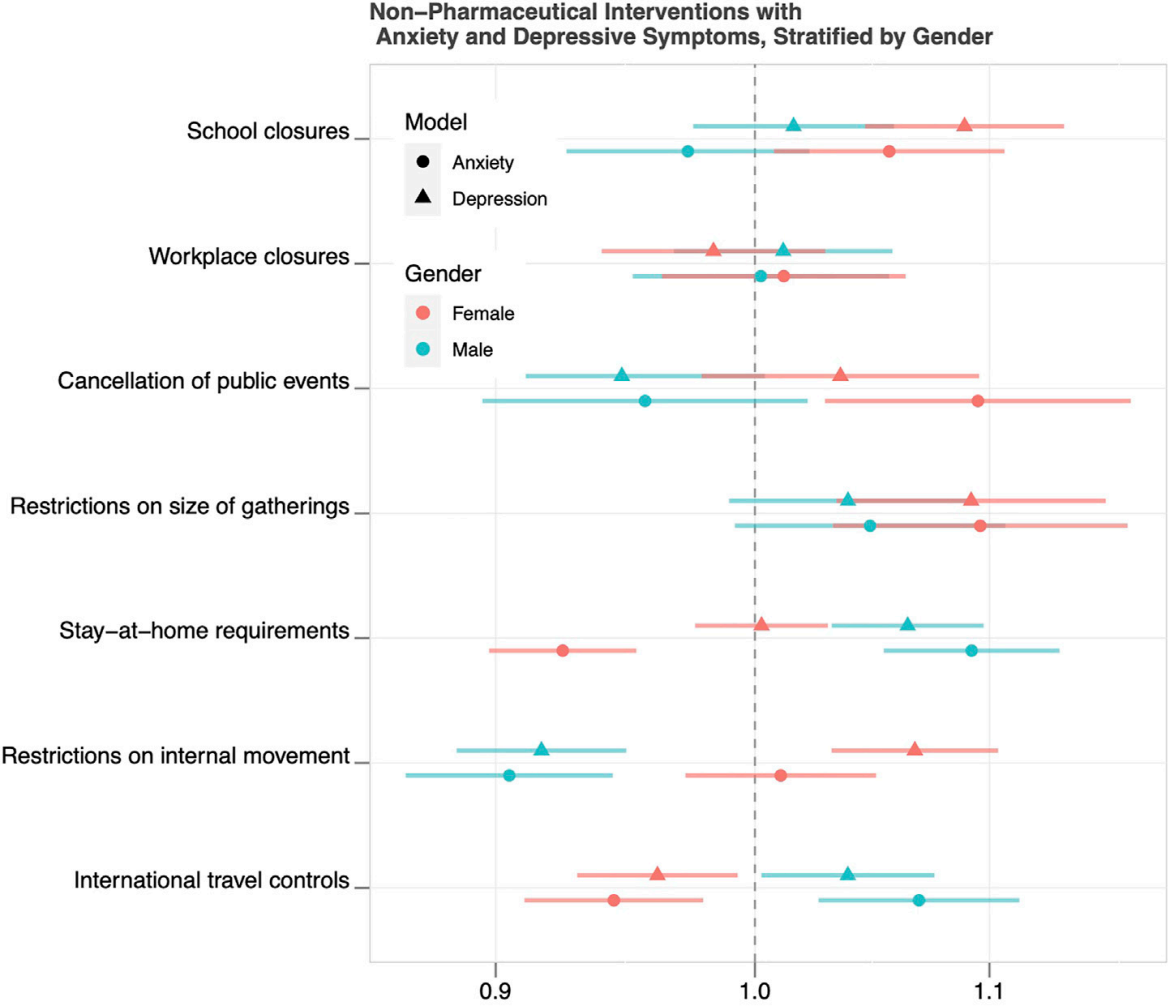
Odds Ratios and 95% Confidence Intervals for Associations of Non-Pharmaceutical Interventions with Anxiety and Depressive Symptoms, Stratified by Gender (43 countries, 2020).

Results of the models with each NPI, and interactions with gender, are displayed in Figure 2. Interactions between each NPI and gender for associations with anxiety and depressive symptoms were statistically significant for every NPI except for workplace closures. Associations of school closures, cancellation of public events, restrictions on the size of gatherings, and restrictions on internal movement with anxiety and depressive symptoms were stronger among females, whereas associations of stay-at-home requirements and international travel controls with anxiety and depressive symptoms were stronger among males. Workplace closures were not associated with either anxiety or depressive symptoms in females or males. The numerical values of these parameter estimates are displayed in Supplementary Table S1. The predicted probabilities of reporting anxiety and depressive symptoms, in the presence and absence of a given NPI, are plotted in Supplementary Figures S5,S6.

**FIGURE 3:**
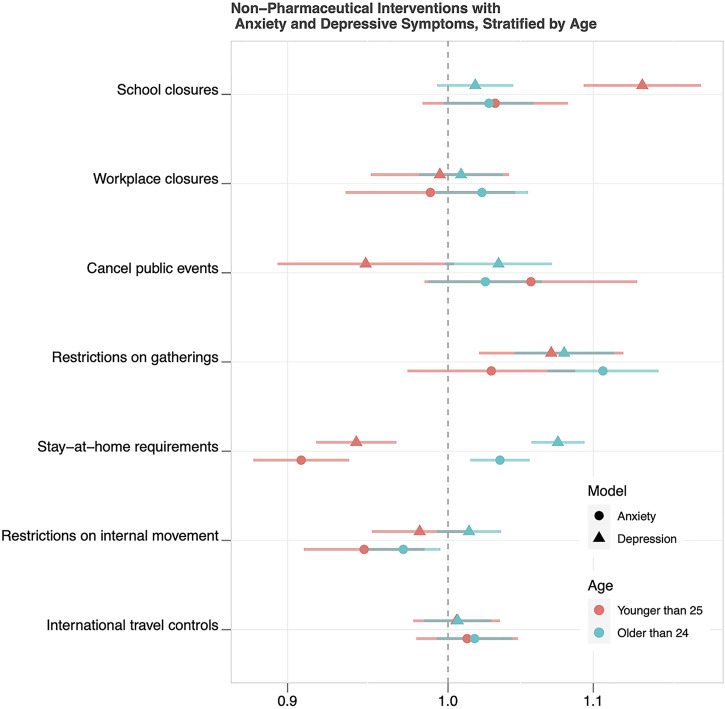
Odds Ratios and 95% Confidence Intervals for Associations of Non-Pharmaceutical Interventions with Anxiety and Depressive Symptoms, Stratified by Age (43 countries, 2020).

Results of the models with each NPI, and interactions with age, are displayed in Figure 3. For anxiety symptoms, interactions between restrictions on the size of gatherings and stay-at-home requirements with age were significant. For depressive symptoms, interactions between school closures, cancellation of public events, stay-at-home requirements, and restrictions on internal movement with age were significant. The numerical values of these parameter estimates are displayed in Supplementary Table S2. The predicted probabilities of reporting anxiety and depressive symptoms, in the presence and absence of a given NPI, are plotted in Supplementary Figures S7,S8.

### Sensitivity Analyses

Results of the univariable models were largely consistent with the pattern of results from the multivariable models (Supplementary Figure S9), suggesting our results were not sensitive to multicollinearity.

## Discussion

In this multi-national study of 43 countries during the COVID-19 pandemic, we found that more stringent implementation of national-level NPIs was associated with higher anxiety and depressive symptoms among adults, even after adjusting for concurrent COVID-19 caseload and mortality. When broken down into the component NPIs, associations were heterogeneous by gender, age, and the type of NPI implemented. Specifically, the association of school closures with anxiety and depressive symptoms was significantly stronger among females compared to males. Patterns of associations among the remaining NPIs were inconsistent by gender; the cancellation of public events, restrictions on the size of gatherings, and restrictions on internal movement were more strongly associated with anxiety and depressive symptoms among females, whereas stronger associations were observed for males for stay-at-home requirements and international travel controls. When stratified by age, associations of NPIs with anxiety and depressive symptoms were less heterogeneous than when stratified by gender, with the most substantial differences observed for stay-at-home requirements.

An important caveat to our findings is that effect sizes for the associations of NPIs with anxiety and depressive symptoms tended to be very small. This is best exemplified in the plots of predicted probabilities of symptoms by policy implementation (Supplementary Figures S3–S8). The complexities of interpreting effect sizes in population health research have been explored in depth elsewhere [32]. In short, effect sizes are likely to be smaller for universal interventions and when the mechanisms of interventions are indirect; these features are plausibly applicable to the NPIs examined in this study. However, given that NPIs operate at the population level, even the small effect sizes observed in our study could translate to measurable and important changes in anxiety and depressive symptoms.

In response to the psychological sequalae of the COVID-19 pandemic, calls have been made for greater mental health surveillance and larger capacity to support public mental health [33–35]. Within these infrastructures, our results provide new information that can assist federal governments in identifying emerging threats to population-level mental health and rapidly responding [36]. There are numerous steps that can be taken by governments to mitigate isolation and loneliness that may be induced by NPIs, and these have been described in depth elsewhere [37–39]. Briefly, possible interventions include media campaigns to promote resiliency and healthy coping; “social prescribing,” or the encouragement of activities such as the arts or physical activity over digital platforms [40]; and the creation of telephone or internet-based hotlines, both for those at high risk of loneliness [41] and those in crisis. In the long-term, building more robust mental health workforces can also assist in the deployment of these interventions in times of crisis [42].

To our knowledge, this is the first study to explore gender and age differences in the association of country-wide NPIs with mental health during the COVID-19 pandemic. School closures were more strongly associated with higher anxiety and depressive symptoms among females compared to males, a finding in line with widespread speculations about the gendered impact of the COVID-19 pandemic and the increased childcare demands due to school closures, which largely fell to women [13, 43]. One recent study found that loss of childcare and participation in homeschooling were associated with increased risk for job loss and reduction in work hours among women but not men [44]. In conjunction with our findings, these patterns reinforce calls for policies that support gender equality in the workplace. Proposed interventions include family-friendly policies (i.e., flexible work hours and part-time programs) that support families with increased childcare responsibilities during the pandemic and expanded childcare tax credits, among others [45]. Additionally, because part-time and casual workers are more likely to be women, existing economic support policies should be broadened to ensure eligibility for unemployment benefits and sick pay [46].

The magnitudes of associations of other NPIs with anxiety and depressive symptoms were also heterogeneous by gender, albeit less consistently than school closures. These differences may be attributable to differences in how males and females perceive stress, attribute the causes of stress, and cope with adversity. For example, in one recent study, endorsing a present-hedonistic time perspective (i.e., “living in the moment”) while under a stay-at-home order was associated with lower depressive symptoms among women, but higher depressive symptoms among men, and this mirrors the pattern of findings seen in our study [47]. Additionally, a well-replicated finding in psychology is that women are more likely to draw on social support systems in times of stress [48]; this could explain why NPIs that restrict access to social circles, including cancellation of public events and restrictions on the size of gatherings, were more strongly associated with anxiety and depressive symptoms among females compared to males. With regards to international travel restrictions, females are less likely to engage in work-related travel [49], and therefore these restrictions may not be as closely tied to mental health as they are for males. Altogether, however, these explanations are speculative, and warrant further exploration to determine more definitive mechanisms.

Many studies have found that young adults have been especially likely to experience poor mental health throughout the COVID-19 pandemic, which some researchers have suggested may be due to age-related vulnerability to the social and individual consequences of certain NPIs [50]. However, in general, we observed less heterogeneity in associations between NPIs and anxiety and depressive symptoms by age than by gender. The largest differences by age were observed for stay-at-home requirements, which were associated with a lower likelihood of anxiety and depressive symptoms among younger adults, but a higher likelihood of anxiety and depressive symptoms among middle-aged and older adults. This finding is somewhat paradoxical, given that younger adults have experienced disproportionate job loss throughout the pandemic [12] and are more likely to live alone [50]. Possible explanations are that younger adults tend to have higher digital literacy [51], which could allow them to leverage virtual platforms for social contact more readily than older adults. Additionally, stay-at-home orders may paradoxically reduce anxiety and depressive symptoms related to exposure to the SARS-CoV-2 virus among essential workers, who are disproportionately likely to be younger adults.

### Limitations

This study has several limitations and strengths. Our results should not be interpreted as indicating that NPIs are causally associated with anxiety and depressive symptoms. When many NPIs are implemented close together in time, which has been the case during the COVID-19 pandemic, isolating their individual effects is exceptionally difficult [52]. Further, we cannot rule out the presence of unmeasured confounding at either the country or individual levels. The stringency index does not capture the extent to which NPIs were adhered to within countries, nor the heterogeneity likely to be present at regional or local levels. Although we included weights to account for survey non-response and to increase representativeness of the general population, the extent to which our sample represents the general population on unmeasured characteristics is unknown. We were unable to locate comparable data on mental health prior to the pandemic to examine historical trends within each country. On the other hand, both our survey and NPI data were temporally granular, enabling us to examine time-lagged associations instead of cross-sectional ones, which contrasts most of the available literature. Additionally, our sample included a large number of adults from 43 nations and spanned over 7 months of the COVID-19 pandemic.

### Conclusion

In summary, we observed heterogeneity in the association of national-level NPIs with anxiety and depressive symptoms by gender and age across 43 countries, supporting the viewpoint that the adverse impacts of the COVID-19 pandemic may be concentrated among certain sociodemographic subgroups. More generally, our results suggest that federal governments around the world should be prepared to address the collateral mental health consequences of NPIs designed to reduce disease transmission in the general population during the COVID-19 pandemic and future health emergencies.
