# Supplementary material for: Association of Non-Pharmaceutical Interventions to Reduce the Spread of SARS-CoV-2 With Anxiety and Depressive Symptoms: A Multi-National Study of 43 Countries
Source: Int J Public Health. 2022 Mar 3;67:1604430. doi: 10.3389/ijph.2022.1604430 (PMC8927027; doi:10.3389/ijph.2022.1604430)
Supplement: Supplementary file 1 [file DataSheet1.docx]

**Supplemental Figure 1.** Number of respondents per week of the study period (43 countries, 2020).

**Supplemental Figure 2.** Kappa Correlations Between Non-Pharmaceutical Interventions (43 countries, 2020).


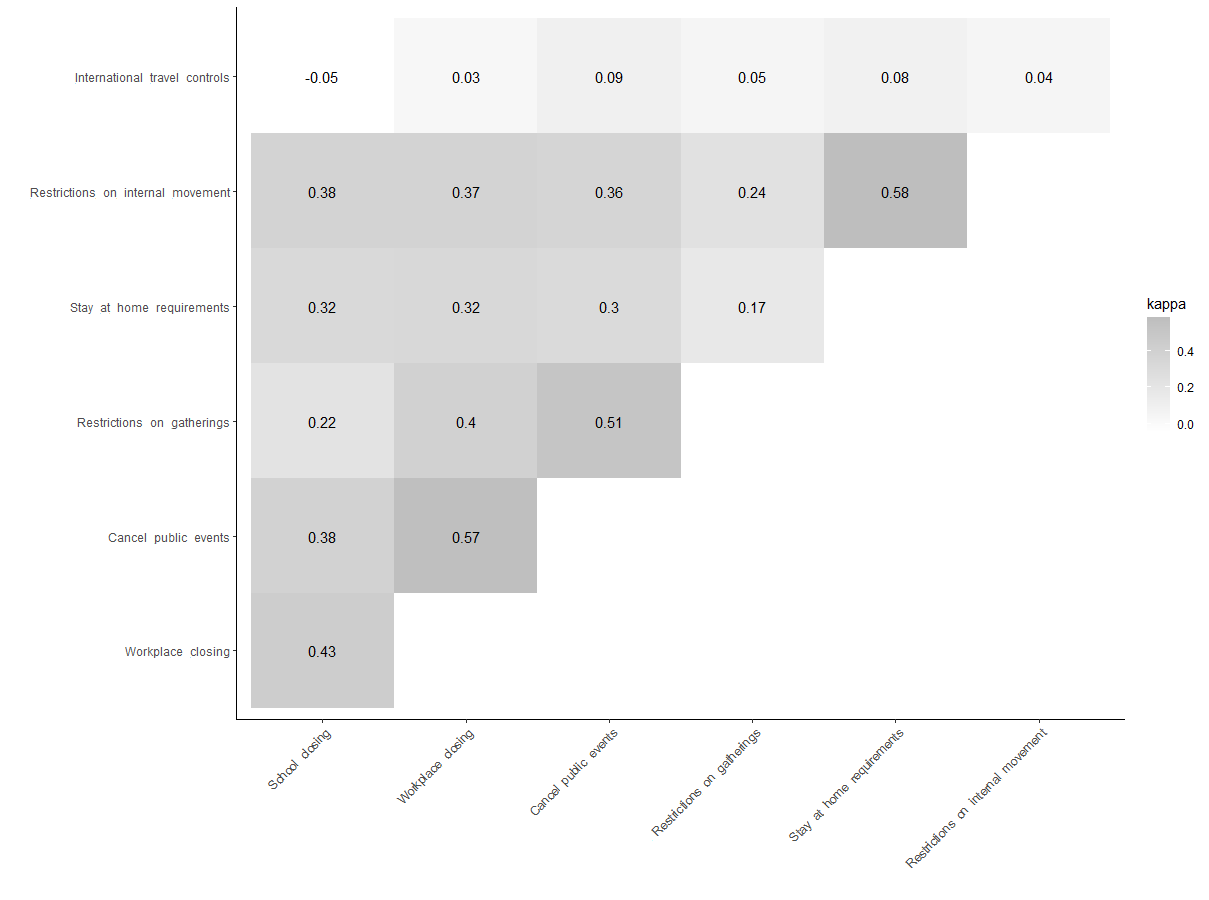


**Supplemental Figure 3.** Probability of Anxiety and Depressive Symptoms by Stringency Policy Score and Gender (43 countries, 2020).


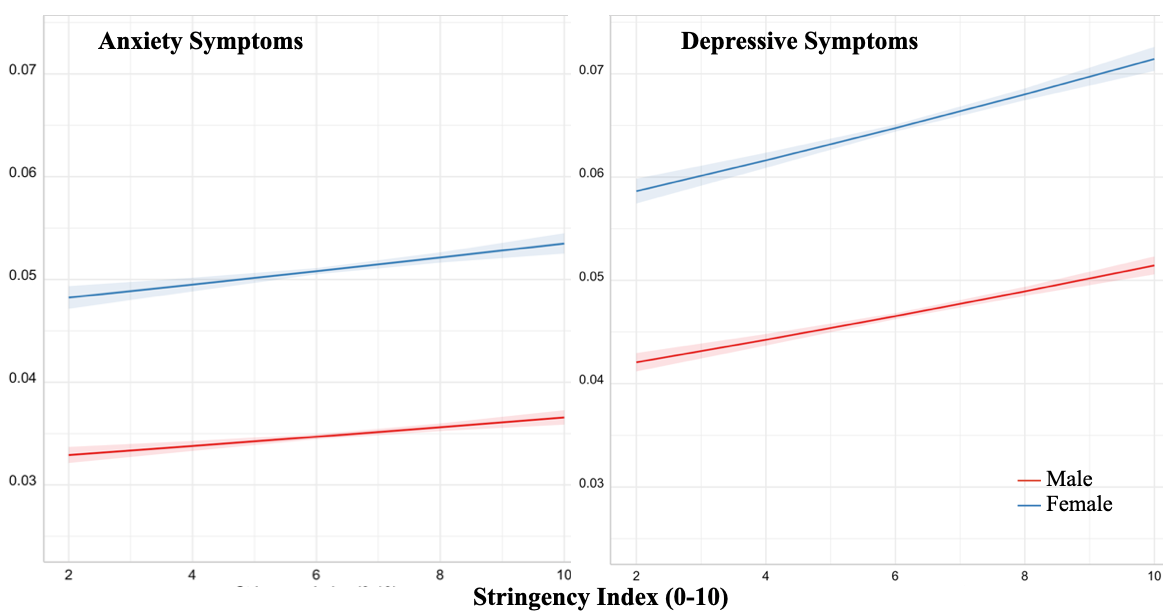


**Supplemental Figure 4.** Probability of Anxiety and Depressive Symptoms by Stringency Policy Score and Age (43 countries, 2020).

**Supplemental Figure 5.** Probability of Anxiety Symptoms by Stringency Policy Component and Gender (43 countries, 2020).

**Supplemental Figure 6.** Probability of Depressive Symptoms by Stringency Policy Component and Gender (43 countries, 2020).

**Supplemental Figure 7.** Probability of Anxiety Symptoms by Stringency Policy Component and Age (43 countries, 2020).

**Supplemental Figure 8.** Probability of Depressive Symptoms by Stringency Policy Component and Age (43 countries, 2020).

**Supplemental Figure 9.** Odds Ratios and 95% Confidence Intervals for Univariable (Single Policy Models) and Multivariable (All Policies Simultaneously) Associations of Non-Pharmaceutical Interventions with Anxiety Symptoms, Stratified by Gender (43 countries, 2020).

**Supplemental Table 1.** Odds Ratios and 95% Confidence Intervals for Associations of Non-Pharmaceutical Interventions with Anxiety and Depressive Symptoms, Stratified by Gender (43 countries, 2020).

| **Anxiety Symptoms** | |  | **CI** | |  |  |
| --- | --- | --- | --- | --- | --- | --- |
|  |  | **OR** | **2.50%** | **97.50%** | **t-val** | **p** |
| **School closures** | Male | 0.973 | 0.926 | 1.022 | -1.598 | 0.771 |
|  | Female | 1.056 | 1.008 | 1.107 | 3.374 | 0.010 |
| **Workplace closures** | Male | 1.002 | 0.952 | 1.056 | 0.133 | 1.000 |
|  | Female | 1.012 | 0.963 | 1.063 | 0.685 | 1.000 |
| **Cancellation of public events** | Male | 0.956 | 0.895 | 1.022 | -1.958 | 0.485 |
|  | Female | 1.095 | 1.029 | 1.165 | 4.223 | 0.000 |
| **Restrictions on size of gatherings** | Male | 1.048 | 0.992 | 1.107 | 2.466 | 0.167 |
|  | Female | 1.096 | 1.032 | 1.164 | 4.430 | 0.000 |
| **Stay-at-home requirements** | Male | 1.092 | 1.054 | 1.132 | 7.143 | 0.000 |
|  | Female | 0.925 | 0.898 | 0.953 | -7.559 | 0.000 |
| **Restrictions on internal movement** | Male | 0.905 | 0.868 | 0.944 | -6.871 | 0.000 |
|  | Female | 1.011 | 0.972 | 1.050 | 0.784 | 0.999 |
| **International travel controls** | Male | 1.069 | 1.026 | 1.113 | 4.729 | 0.000 |
|  | Female | 0.944 | 0.911 | 0.979 | -4.572 | 0.000 |

| **Depression Symptoms** | |  | **CI** | |  |  |
| --- | --- | --- | --- | --- | --- | --- |
|  |  | **OR** | **2.50%** | **97.50%** | **t-val** | **p** |
| **School closures** | Male | 1.016 | 0.975 | 1.058 | 1.112 | 0.979 |
|  | Female | 1.089 | 1.046 | 1.134 | 6.102 | 0.000 |
| **Workplace closures** | Male | 1.012 | 0.968 | 1.057 | 0.749 | 1.000 |
|  | Female | 0.983 | 0.940 | 1.029 | -1.075 | 0.984 |
| **Cancellation of public events** | Male | 0.947 | 0.894 | 1.004 | -2.694 | 0.091 |
|  | Female | 1.035 | 0.979 | 1.095 | 1.785 | 0.626 |
| **Restrictions on size of gatherings** | Male | 1.039 | 0.990 | 1.090 | 2.266 | 0.268 |
|  | Female | 1.092 | 1.034 | 1.153 | 4.653 | 0.000 |
| **Stay-at-home requirements** | Male | 1.064 | 1.032 | 1.097 | 5.823 | 0.000 |
|  | Female | 1.003 | 0.976 | 1.030 | 0.287 | 1.000 |
| **Restrictions on internal movement** | Male | 0.917 | 0.886 | 0.949 | -7.285 | 0.000 |
|  | Female | 1.067 | 1.032 | 1.104 | 5.560 | 0.000 |
| **International travel controls** | Male | 1.038 | 1.003 | 1.076 | 3.108 | 0.026 |
|  | Female | 0.961 | 0.930 | 0.993 | -3.519 | 0.006 |

**Supplemental Table 2.** Odds Ratios and 95% Confidence Intervals for Associations of Non-Pharmaceutical Interventions with Anxiety and Depressive Symptoms, Stratified by Age (43 countries, 2020).

| **Anxiety Symptoms** | |  | **CI** | |  |  |
| --- | --- | --- | --- | --- | --- | --- |
|  |  | **OR** | **2.50%** | **97.50%** | **t-val** | **p** |
| **School closures** | 18-24 | 1.031 | 0.983 | 1.082 | 1.879 | 0.552 |
|  | ≥ 25 | 1.027 | 0.997 | 1.058 | 2.646 | 0.104 |
| **Workplace closures** | 18-24 | 0.988 | 0.935 | 1.045 | -0.604 | 1.000 |
|  | ≥ 25 | 1.022 | 0.992 | 1.054 | 2.124 | 0.362 |
| **Cancellation of public events** | 18-24 | 1.056 | 0.985 | 1.132 | 2.257 | 0.275 |
|  | ≥ 25 | 1.025 | 0.987 | 1.064 | 1.908 | 0.529 |
| **Restrictions on size of gatherings** | 18-24 | 1.029 | 0.974 | 1.087 | 1.495 | 0.842 |
|  | ≥ 25 | 1.107 | 1.067 | 1.148 | 8.062 | 0.000 |
| **Stay-at-home requirements** | 18-24 | 0.908 | 0.880 | 0.937 | -8.877 | 0.000 |
|  | ≥ 25 | 1.035 | 1.015 | 1.055 | 5.065 | 0.000 |
| **Restrictions on internal movement** | 18-24 | 0.946 | 0.910 | 0.985 | -4.025 | 0.001 |
|  | ≥ 25 | 0.971 | 0.948 | 0.995 | -3.482 | 0.007 |
| **International travel controls** | 18-24 | 1.013 | 0.979 | 1.047 | 1.084 | 0.984 |
|  | ≥ 25 | 1.018 | 0.993 | 1.043 | 2.032 | 0.430 |

| **Depression Symptoms** | |  | **CI** | |  |  |
| --- | --- | --- | --- | --- | --- | --- |
|  |  | **OR** | **2.50%** | **97.50%** | **t-val** | **p** |
| **School closures** | 18-24 | 1.136 | 1.093 | 1.181 | 9.580 | 0.000 |
|  | ≥ 25 | 1.018 | 0.993 | 1.044 | 2.071 | 0.401 |
| **Workplace closures** | 18-24 | 0.995 | 0.951 | 1.041 | -0.342 | 1.000 |
|  | ≥ 25 | 1.009 | 0.981 | 1.037 | 0.900 | 0.997 |
| **Cancellation of public events** | 18-24 | 0.947 | 0.894 | 1.004 | -2.706 | 0.088 |
|  | ≥ 25 | 1.034 | 0.998 | 1.071 | 2.739 | 0.080 |
| **Restrictions on size of gatherings** | 18-24 | 1.070 | 1.021 | 1.122 | 4.141 | 0.000 |
|  | ≥ 25 | 1.079 | 1.045 | 1.115 | 6.760 | 0.000 |
| **Stay-at-home requirements** | 18-24 | 0.942 | 0.917 | 0.967 | -6.616 | 0.000 |
|  | ≥ 25 | 1.075 | 1.056 | 1.094 | 11.963 | 0.000 |
| **Restrictions on internal movement** | 18-24 | 0.982 | 0.951 | 1.013 | -1.715 | 0.687 |
|  | ≥ 25 | 1.014 | 0.993 | 1.035 | 1.889 | 0.545 |
| **International travel controls** | 18-24 | 1.006 | 0.977 | 1.035 | 0.572 | 1.000 |
|  | ≥ 25 | 1.006 | 0.984 | 1.029 | 0.831 | 0.999 |

**Supplemental Text**

Countries included in analyses: Argentina, Australia, Austria, Belgium, Brazil, Bulgaria, Canada, Chile, Colombia, Costa Rica, Czech Republic, Denmark, Estonia, Finland, France, Germany, Greece, Hungary, Iceland, India, Indonesia, Ireland, Israel, Italy, Japan, Latvia, Lithuania, Luxembourg, Mexico, Netherlands, New Zealand, Norway, Poland, Portugal, Romania, Russia, Slovenia, South Africa, Spain, Sweden, Switzerland, Turkey, United Kingdom
